# A genetic variant in miR‐100 is a protective factor of childhood acute lymphoblastic leukemia

**DOI:** 10.1002/cam4.2082

**Published:** 2019-03-07

**Authors:** Yao Xue, Xiaoyun Yang, Shaoyan Hu, Meiyun Kang, Jing Chen, Yongjun Fang

**Affiliations:** ^1^ Department of Hematology and Oncology Children’s Hospital of Nanjing Medical University Nanjing China; ^2^ Key Laboratory of Hematology Nanjing Medical University Nanjing China; ^3^ Department of Hematology and Oncology Soochow University Affiliated Children’s Hospital Suzhou China; ^4^ Key Laboratory of Pediatric Hematology and Oncology Ministry of Health, Department of Hematology and Oncology, Shanghai Children’s Medical Center Shanghai Jiao Tong University School of Medicine Shanghai China

**Keywords:** childhood ALL, genetic variants, miRNA, susceptibility

## Abstract

**Background:**

In the past decade, miR‐100, miR‐146a, and miR‐210 were reported to be dysregulated in childhood acute lymphoblastic leukemia (ALL). However, effects of genetic variants in these three microRNAs have not been investigated in Chinese population.

**Methods:**

In this study, we conducted a case‐control study to evaluate the relationship between genetic variants in *miR‐100, miR‐146a,* and *miR‐210* and the risk of childhood ALL in Chinese population. Subsequently, plasma expression level of miR‐100 was also detected.

**Result:**

We found that subjects carrying mutant homozygous TT genotype of *miR‐100* rs543412 had a statistically significantly decreased risk of childhood ALL (adjusted odds ratio [OR] = 0.73, 95% confidence interval [CI] = 0.55‐0.97, *P* = 0.029). This protective effect was also observed among subjects whose parents were ever drinkers (adjusted OR = 0.53, 95% CI = 0.29‐0.94), or whose living house were ever painted (adjusted OR = 0.57, 95% CI = 0.34‐0.94). Besides, rs543412 variant homozygous TT had a significantly protective role in patients with childhood B‐ALL. Finally, we found that expression level of miR‐100 in plasma of childhood ALL cases was significantly higher than that of noncancer controls.

**Conclusion:**

Our study suggested that there was significant association between the polymorphisms in *miR‐100* (rs543412) and decreased susceptibility to childhood ALL.

## 1 BACKGROUND

Acute lymphoblastic leukemia (ALL) is the most common malignant disease in childhood. The incidence of childhood ALL has been slowly increasing since 1975 worldwide.^1^ This disease was manifested by an uncontrolled expansion of immature lymphocytes to all over the body. Over the past few decades, great advances has been made in the treatment of pediatric ALL and has remarkably increased its 5‐year survival rates, approximately 85% of patients aged 1‐18 years with newly diagnosed ALL treated on current regimens are expected to be long‐term event‐free survivors, with over 90% surviving at 5 years.^2^ Despite these improvements, ALL was still a serious threat to childhood life. Previous studies have indicated that genetic variants significantly influence the susceptibility of childhood ALL.^3^

MicroRNAs (miRNAs) are a class of short noncoding RNAs that regulate target gene expression by binding to 3′‐untranslated region of mRNA.^4^ Numbers of miRNAs have been described to participate in almost all human cancers,^5^, ^6^ indicating the important role of miRNAs in carcinogenesis. Among them, we noticed some reviews focused on miRNAs in childhood ALL have mentioned miR‐100, miR‐210, and miR‐146a in common.^8^, ^9^ In our further literation retrieval, miR‐100,^10^, ^11^ miR‐146a,^12^, ^13^ as well as miR‐210^14^, ^15^ were all reported to have remarkable significance in ALL for several times, especially in pediatric ALL. For example, the restoration of miR‐100 in ALL cells suppressed cell proliferation and increased dexamethasone‐induced cell apoptosis,^16^ while miR‐146a was reported to be highly expressed in pediatric ALL bone marrow samples.^17^ Studies have provided multi‐level evidence for the biological effects of these three miRNAs in pathogenesis and development in childhood ALL.

In the past decade, researches have shown that genetic variations in miRNAs can attenuate or even deprive their function of regulating target genes expression. Therefore, polymorphisms in miRNAs were significantly associated with the susceptibility to various cancers, including childhood ALL.^18^, ^19^ Therefore, it is rationally to conjecture that genetic variants in *miR‐100*, *miR‐146a,* and *miR‐210* may have potential effects on susceptibility of childhood ALL. However, relative investigation has not been reported in Chinese population.

To test this hypothesis, we searched for genetic variants located in these three miRNAs, and investigated whether they were associated with susceptibility of childhood ALL in Chinese population by a case‐control study.

## 2 MATERIALS AND METHODS

### 2.1 Study population

Recruitment criterion of subjects has been described previously.^21^ All patients had been diagnosed as ALL by morphology, immunology, cytogenetic and molecular biology in Children’s Hospital of Nanjing Medical University, Soochow University Affiliated Children’s Hospital, and Shanghai Children’s Medical Center from January 2007 to October 2016. Control subjects were recruited from the same geographic area at the same period of time with cases. The present study samples were expended group of the previously described.^21^ However, as the biological sample bank has been established for many years, and some of the DNA samples collected during early years have been used up, so some samples recruited in the previous study were not included now. Besides, we have added some new samples in recent years. Therefore, there are some differences between observed samples in these two articles. Because some of the biological samples were not successfully genotyped in the experiment, finally 831 childhood ALL patients and 1 079 cancer‐free controls were analyzed in our present study. Controls were enrolled as never had malignant neoplasm or hematological diseases. All recruited subjects were Han Chinese aged 0‐18 years old, and had no genetic relationship with each other. Exclusion standard of cases were who had been diagnosed with other hematological disorders or cancers. Blood samples were taken from each subject after informed consent was obtained from the parents. In addition, we obtained demographic and environmental exposure information about the subjects using questionnaires. For environmental exposure factors, we used smoking and drinking by the parents and house painting. If neither father nor mother of the subject was a smoker, the question was marked “never”; otherwise it was marked “ever.” If neither father nor mother of the subject was a drinker, the question was defined as “never”; otherwise it was defined as “ever.” If the house was painted during pregnancy or after birth, the house painting status was marked “ever”; otherwise it was marked “never.” In addition, plasma samples were obtained from another 88 childhood ALL cases as well as 99 cancer‐free controls. The research protocol was approved by the Medical Ethics Committee of Children’ Hospital of Nanjing Medical University. All the guardians of participants signed an informed consent for participation in this study.

### 2.2 Selection of genetic variation

Genetic variants (ie, single nucleotide polymorphisms, SNPs) located in 500 bp upstream to 500 bp downstream of miRNA gene regions were screened by NCBI Variation Viewer (https://www.ncbi.nlm.nih.gov/variation/view/). Minor allele frequency (MAF) in Chinese population of >0.05 was set as inclusion criteria of SNPs. Finally, three SNPs (ie, rs543412, rs2910164, and rs7395206) were selected in our present study, located in upstream of *miR‐100*, gene region of *miR‐146a* and downstream of *miR‐210*, respectively.

### 2.3 Genotyping

Genomic DNA was isolated from peripheral blood lymphocytes following standard protocols of QIAamp DNA Blood Mini Kit (Qiagen, Beijing, China). Genotypes of the 3 SNPs were detected using the SNaPshot (SNaPshot kit, ABI) detection, which was conducted by Biohelper Company (Nanjing, China). Amplification primers are listed in Table S1. Approximately 10% of the samples were randomly selected by a double‐blind method for repeat experiments and the results were 100% concordant.

### 2.4 Expression level of miRNA in plasma

We extracted RNA from 200 μL plasma of 88 ALL cases and 99 cancer‐free controls using QIAGEN miRNeasy Mini Kit (Qiagen, Valencia, CA). The RNA was dissolved by 30 μL sterile water. We used 6 μL RNA to synthesize double‐stranded cDNA by Vazyme HIScript Q Select RT SuperMix (Vazyme, Nanjing, China) for RNA reverse transcription in a 15 μL reaction volume. qRT‐PCR was performed using AceQ qPCR SYBR Green Master Mix (Low ROX Premixed) (Vazyme) by QuantStudio 3. The primer was provided by Bulge‐Loop™ miRNA qRT‐PCR Primer (one RT primer and a pair of q‐PCR primers specific for miR‐100, RIBOBIO, Guangzhou, China). We used Micro‐16‐5p to normalize the data set. The relative expression was calculated by the 2‐ΔΔCt method.

### 2.5 Statistical analyses

Chi‐square test was used to compare the differences in frequency distributions of selected demographic variables, environmental factors, as well as each allele and genotypes of the selected SNPs between cases and controls. Multivariate logistic regression analyses were performed to calculate the adjusted odds ratios (ORs) for estimating risk of ALL and their 95% confidence intervals (CIs). The multivariate adjustment included age, gender, parental smoking status, parental drinking status, and house painting status. Stratification analysis was conducted according to different subgroups of age, gender and immune‐phenotype. Hardy‐Weinberg equilibrium of the genotype distribution among the control group was tested using a goodness‐of‐fit chi‐square test. Differences in expression levels of miR‐100 between plasma of cases and controls were evaluated by an independent sample t test. All statistical tests were two‐sided at a significance level of 0.05 and were analyzed using the SAS software (version 9.1.3; SAS Institute, Cary, NC) unless otherwise indicated.

## 3 RESULTS

### 3.1 Characteristics of the study subjects

The distributions of selected characteristics of the 831 childhood ALL patients and 1 079 controls are presented in Table 1. The cases and controls were adequately matched for age and gender (*P* = 0.123 for age and 0.288 for gender). As for environmental exposures, distribution frequency of parental smoking status was almost the same between cases and control groups (*P* = 0.978). However, we found that parental drinking status was a significant risk factor for childhood ALL (*P* = 0.003). Furthermore, as expected, there were more cases with house painted (37.1%) than controls (26.2%), and the difference was significant (*P < *0.001). As for clinical subgroups, patients with B‐ALL were in the majority (87.6%).

**Table 1:** Frequency distribution of selected variables between cases with childhood ALL and cancer‐free controls

Variables	Cases (*n* = 831)		Controls (*n* = 1079)		*P* a
	*n*	%	*n*	%	
Age (years)					
≤5	460	55.4	559	51.8	0.123
>5	371	44.6	520	48.2	
Gender					
Male	493	59.3	666	61.7	0.288
Female	338	40.7	413	38.3	
Parental smoking status					
Never	391	47.1	507	47.0	0.978
Ever	440	52.9	572	53.0	
Parental drinking status					
Never	613	73.8	858	79.5	**0.003**
Ever	218	26.2	221	20.5	
House—painting status					
Never	523	62.9	796	73.8	**＜0.001**
Ever	308	37.1	283	26.2	
Immunophenotype					
B‐ALL	728	87.6			
T‐ALL	99	11.9			
Others^b^	4	0.5			

Bold values indicated significant differences with *P* < 0.05.

ALL, acute lymphoblastic leukemia.

a Two‐sided chi‐square test for the frequency distribution of selected variables between ALL cases and cancer‐free controls.

b Four patients were ALL with T/B biphenotype.

### 3.2 Genotype distribution in study subjects

Hardy‐Weinberg equilibrium was observed for genotypes distribution among the controls. The genotype distribution of the three SNPs in cases and controls are presented in Table 2. As a result, we found that subjects carrying mutant homozygous TT genotype of rs543412 had a statistically significantly decreased risk of ALL (adjusted OR = 0.73, 95% CI = 0.55‐0.97, *P* = 0.029), when compared with wide homozygous CC carriers. However, similar association was not found in heterozygous CT carriers (adjusted OR = 0.90, 95% CI = 0.73‐1.10, *P* = 0.286), and combined variant genotypes carriers (adjusted OR = 0.85, 95% CI = 0.71‐1.03, *P* = 0.100) of rs543412. In addition, rs543412 mutant T allele frequency was also significantly different between case and control groups (37.2% vs 40.5%, *P* = 0.035). As for rs2910164 and rs7395206, no significant association with susceptibility of childhood ALL were observed in our study subjects.

**Table 2:** Genotype and allele frequencies of the selected single nucleotide polymorphisms among the cases and controls and the associations with childhood acute lymphoblastic leukemia risk

Genotypes	Cases		Controls		Adjusted OR (95% CI)^a^	*P*‐value^b^
	*n*	%	*n*	%		
Rs543412	831	100.0	1079	100.0		0.095
CC	324	39.0	382	35.4	1.00	
CT	396	47.7	519	48.1	0.90 (0.73‐1.10)	0.286
TT	111	13.3	178	16.5	**0.73 (0.55‐0.97)**	**0.029**
CT/TT	507	61.0	697	64.6	0.85 (0.71‐1.03)	0.100
T allele		37.2		40.5		**0.035**
Rs2910164	831	100.0	1079	100.0		0.483
CC	263	31.7	369	34.2	1.00	
CG	429	51.6	541	50.1	1.12 (0.91‐1.37)	0.296
GG	139	16.7	169	15.7	1.20 (0.91‐1.58)	0.206
CG/GG	568	68.3	710	65.8	1.14 (0.93‐1.38)	0.206
G allele		42.5		40.7		0.261
Rs7395206	831	100.0	1079	100.0		0.843
TT	386	46.5	515	47.7	1.00	
CT	353	42.5	450	41.7	1.04 (0.86‐1.27)	0.683
CC	92	11.0	114	10.6	1.09 (0.80‐1.48)	0.590
CT/CC	445	53.5	564	52.3	1.05 (0.87‐1.26)	0.607
C allele		32.3		31.4		0.557

Bold values indicated significant differences with *P* < 0.05.

CI, confidence interval; OR, odds ratio.

a Adjusted for age, gender, smoking status, drinking status, and painting status in logistic regression model.

b Two‐sided χ^2^ test for genotype and allele distributions between the cases and controls.

In further analysis stratified by demographic information and environmental exposure of study subjects (Table 3), we found a more pronounced association between the decreased ALL risk and rs543412 TT genotype among subjects whose parents were ever drinkers (adjusted OR = 0.53, 95% CI = 0.29‐0.94), or whose living house were ever painted (adjusted OR = 0.57, 95% CI = 0.34‐0.94). However, there was no interaction effect observed between environmental exposure and rs543412 genotype. Further studies with larger sample size are needed to detect the interaction effect.

**Table 3:** Stratification analysis of association between rs543412 and childhood acute lymphoblastic leukemia by demographic factors and environmental exposure

Variables	Cases/controls	Genotypes (cases/controls)			Adjusted OR (95% CI)^a^		
		CC	CT	TT	CC	CT	TT
Age (years)							
≤5	460/559	179/196	220/268	61/95	1.00	0.90 (0.68‐1.18)	0.69 (0.47‐1.02)
>5	371/520	145/186	176/251	50/83	1.00	0.90 (0.67‐1.21)	0.76 (0.50‐1.16)
Gender							
Male	493/666	179/235	249/319	65/112	1.00	1.00 (0.77‐1.29)	0.74 (0.51‐1.07)
Female	338/413	145/147	147/200	46/66	1.00	0.77 (0.56‐1.05)	0.69 (0.44‐1.07)
Parental smoking status							
Never	391/507	159/179	179/252	53/76	1.00	0.80 (0.60‐1.06)	0.78 (0.52‐1.19)
Ever	440/572	165/203	217/267	58/102	1.00	1.01 (0.76‐1.33)	0.69 (0.47‐1.02)
Interaction (multiplicative)					*P* b = 0.922		
Parental drinking status							
Never	613/858	235/308	294/415	84/135	1.00	0.92 (0.73‐1.16)	0.82 (0.60‐1.14)
Ever	218/221	89/74	102/104	27/43	1.00	0.81 (0.54‐1.23)	**0.53 (0.29‐0.94)**
Interaction (multiplicative)					*P* c = 0.197		
House—painting status							
Never	523/796	208/282	241/390	74/124	1.00	0.84 (0.66‐1.06)	0.81 (0.58‐1.14)
Ever	308/283	116/100	155/129	37/54	1.00	1.03 (0.71‐1.47)	**0.57 (0.34‐0.94)**
Interaction (multiplicative)					*P* d = 0.583		

Bold values indicated significant differences with *P* < 0.05.

CI, confidence interval; OR, odds ratio.

a Adjusted for age, gender, smoking status, drinking status, and painting status in logistic regression model.

b Adjusted for age, gender, drinking status, and painting status.

c Adjusted for age, gender, smoking status, and painting status.

d Adjusted for age, gender, smoking status, and drinking status.

**Table 4:** Stratification analysis of association between rs543412 and childhood ALL by clinical feature

Variables	Genotypes (cases/controls^b^)			Adjusted OR (95% CI)^a^		
	CC	CT	TT	CC	CT	TT
Immunophenotype						
B‐ALL	278/382	355/519	95/178	1.00	0.95 (0.77‐1.17)	**0.73 (0.54‐0.99)**
T‐ALL	45/382	38/519	16/178	1.00	**0.62 (0.40‐0.99)**	0.77 (0.42‐1.42)

Bold values indicated significant differences with *P* < 0.05.

ALL, acute lymphoblastic leukemia; CI, confidence interval; OR, odds ratio.

a Adjusted for age, gender, smoking status, drinking status and painting status in logistic regression model.

b Number of controls with different genotypes under each subgroup of clinical feature represented all the controls with each genotype.

In stratification analysis according to clinical features, results showed that rs543412 variant homozygous TT had a significantly protective role in patients with childhood B‐ALL (adjusted OR = 0.73, 95% CI = 0.54‐0.99), compared with CC genotypes. In addition, carriers of CT genotype had a remarkable decreased risk of childhood ALL in T‐ALL subgroups (adjusted OR = 0.62, 95% CI = 0.40‐0.99) (Table 4).

### 3.3 Plasma expression level of miR‐100

**Figure 1:**
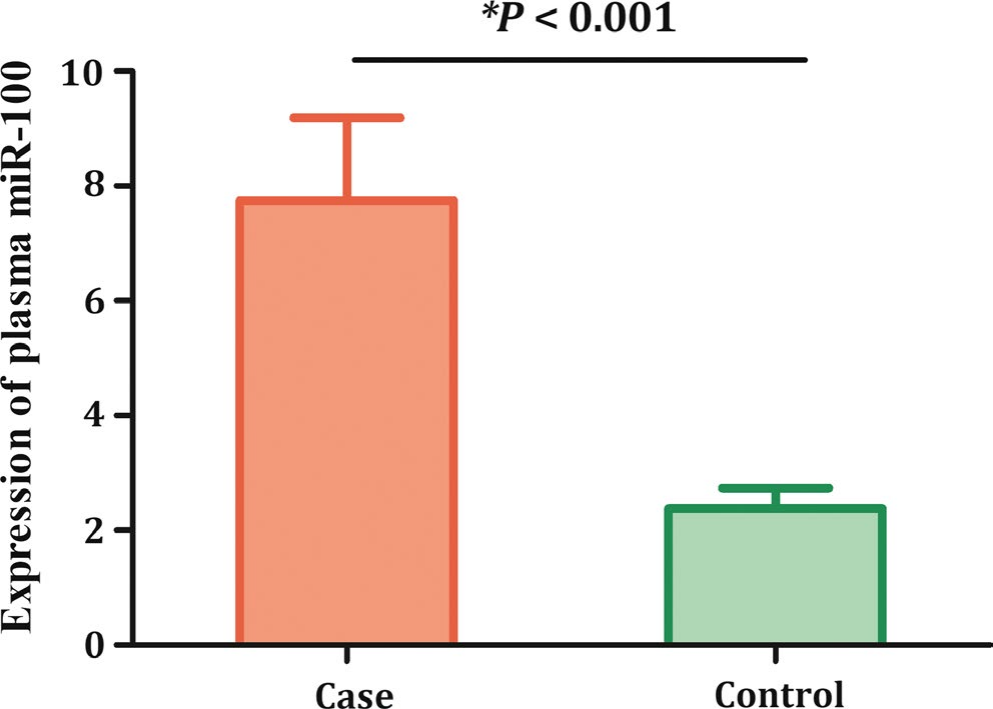
Expression level of miR‐100 in plasma was significantly higher in acute lymphoblastic leukemia (ALL) cases than that in controls

**Figure 2:**
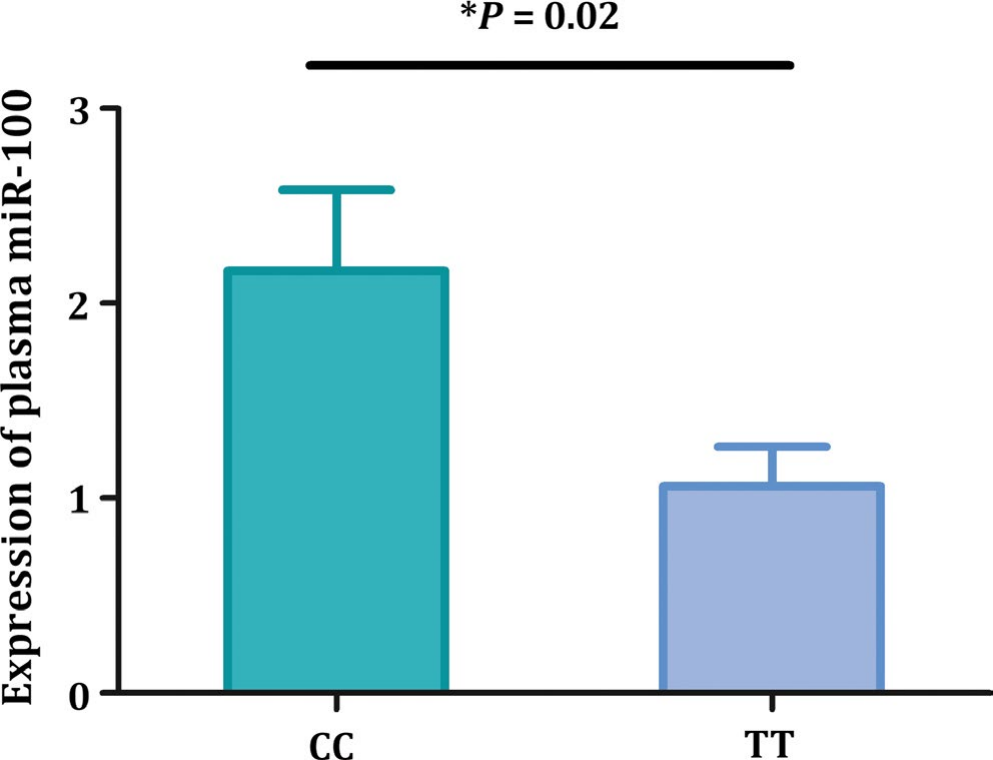
Expression level of miR‐100 in plasma was significantly lower in subjects carrying mutant TT genotype than that with wild CC genotype

As genetic variant rs543412 was located in upstream of *miR‐100*, we subsequently detected the expression level of miR‐100 in plasma of 88 childhood ALL cases as well as 99 non‐cancer controls. As shown in Figure 1, remarkable higher expression level of miR‐100 was observed in ALL cases (*P* < 0.001, fold change = 3.25), providing further evidence for biological role of miR‐100 in this disease. In addition, we detected association between genotype of rs543412 and plasma miR‐100 level in the cancer free controls. As genetic analyses revealed significance of TT genotype in susceptibility of childhood ALL, we evaluated miR‐100 plasma level of subjects carrying TT genotype, compared with wild CC genotype especially. Result is shown in Figure 2. We found that individuals with TT genotype have a significantly lower level of miR‐100.

## 4 DISCUSSION

Nowadays, many studies have focused on abnormal expression level of miRNAs in ALL in that this maybe an indication of important function of miRNAs in pathogenesis of ALL.^22^, ^23^ Meanwhile, genetic variants located in or near miRNA genes were also striking for their function of influencing miRNA binding capacity or expression level.^24^, ^25^ Through our literature review, we found some influential review focused on miRNAs in childhood ALL have mentioned miR‐100, miR‐210, and miR‐146a in common.^8^, ^9^ In the present study, we focused on genetic variants of *miR‐100*, *miR‐146a*, and *miR‐210*, which were all demonstrated to be participate in carcinogenesis of ALL,^13^, ^14^, ^16^ and investigated their association with susceptibility of childhood ALL. Among them, rs7395206 have never been reported in other studies while rs543412 and rs2910164 has not been detected in Chinese childhood ALL groups.

Firstly, our present study found that parental drinking status and house painting was a significant risk factor for childhood ALL, which was consistent with previous studies.^26^, ^27^ It may be interpreted that alcohol may induce reductions in sperm cytosine methyl transferase messenger RNA levels by reducing DNA methylation and disturbing folate metabolism.^28^, ^29^ In addition, house painting may cause damage to reproductive cells of parents or to hemopoietic cells of children themselves, all of these lead to development of childhood leukemia.^27^

To date, several studies about childhood ALL has demonstrated important role of miR‐100. Firstly, it was reported to be approximately 20‐fold up‐regulation in pediatric ALL cases resistance to vincristine and daunorubicin,^30^ and was also distinguished expressed in TEL/AML1 ALL subgroups, this will lead to good outcomes. Coincidently, Oliveira et al found that higher miR‐100 expression was associated with presence of 12;21 translocation (ie, TEL/AML1 fusion gene), lower level of white blood cells at diagnosis (<50 000/mm^3^), and hyperdiploid negative in pediatric ALL subjects.^10^ These observation provided evidence for involvement of miR‐100 in underlying biology of childhood ALL. For the detailed molecular mechanism of miR‐100 in ALL, Li et al reported that miR‐100 played critical roles in altering cellular processes by targeting both the FKBP51 and IGF1R/mTOR signaling pathways.^16^ However, after retrieving literatures in PubMed, we did not find studies about association between genetic variants in *miR‐100* gene and ALL. We only found Jana et al have investigate MAF of rs543412 (in miR‐100) in chronic lymphocytic leukemia (CLL) in European population and reported that frequency of alternative allele T was significantly lower in CLL patients than that in 1 000 genomes project.^31^ In our present study, we searched for SNPs located in *miR‐100* gene region by NCBI Variation Viewer and also selected rs543412. This genetic variation located within 500bp downstream of *miR‐100* gene and was theoretically capable of influencing miR‐100 expression. The current study revealed that mutant homozygous TT genotype of rs543412 had a remarkable protective role of childhood ALL. For the past decades, researchers have demonstrated various functional mechanism of genetic variant in carcinogenesis, such as promoter regulation, mRNA splicing, transcriptional alternation et al. As for SNPs located nearby miRNA genes, previous studies reported that they may interfere with the mature processing or the degradation of miRNA by compromising the secondary structure and cause a dysregulation of the amount of mature miRNA.^32^ Therefore we inferred that rs543412 TT genotype may influence susceptibility of ALL through alternating miR‐100 expression levels and further studies investigating biological function of this SNP is still required. Furthermore, we also found that rs543412 TT genotype was notably associated with decreased risk of ALL in children subjects whose parents were ever drinkers, or whose living house were ever painted. Alcohol exposure and house painting may cause cell damage through specific biological process, such as DNA methylation, folate metabolism.^27^, ^28^ It may be interpreted that miR‐100 may have potential role in alcohol or decoration pollution related cell damage process, hence the effect of miR‐100 polymorphism may be more remarkable in subgroups of subjects with parental‐drinker or house painted.

In addition, in our study, expression level of miR‐100 in plasma of ALL cases was significantly higher than that of controls, which was not accordant with previous findings^10^ that miR‐100 level was lower in ALL bone marrow samples compared to control samples. We inferred that the discrepancy may be related with different biological characteristic between plasma samples and bone marrow samples, and may also arise from different study samples. Our results implicate that genetic variant of miR‐100 was notably associated with decreased childhood ALL risk, and the plasma miR‐100 expression level was also different in ALL cases from that in controls. Taken together, these observations supported an important role of miR‐100 in carcinogenesis of childhood ALL in both genetic and transcriptional levels. As plasma sample was relatively easy to be obtained in clinical practice, our present observation suggested that miR‐100 maybe a potential biomarker of childhood ALL.

Substantial studies have demonstrated that miR‐146a can act as a tumor suppressor or oncogene in various human cancers, such as gastric cancer,^33^ breast cancer,^34^ lung cancer,^35^ etc. It was shown to be a critical regulator of hematopoietic stem cells (HSCs) homeostasis during chronic inflammation and deletion of miR‐146a leaded to HSCs exhaustion and hematopoietic neoplasms.^36^ In research field of ALL, Saki et al reported that ectopic expression of miR‐146a resulted in significant up‐regulation of CCAAT/enhancer‐binding protein alpha and GATA3 in Jurkat T cells.^37^ Moreover, genetic variants of *miR‐146a* (ie, rs2910164) have also shown to be associated with risk of multiple cancers.^38^, ^39^ In 2014, Hasani et al have demonstrated that rs2910164 was associated with increased risk of pediatrics ALL in an Iranian population.^40^ However, in our present study, no significant association was found between rs2910164 and risk of childhood ALL, we inferred that this inconsistency may be raised from different population race.

In this study, we have also investigated the association between genetic variants in *miR‐210* and risk of pediatric ALL. However, there was no remarkable association observed. Although miR‐210 has been reported to be participate in childhood ALL,^14^ our result suggested that SNPs located in *miR‐210* may not exert biological effect on pathogenesis of this disease.

In conclusion, our study aimed to analyze association between the polymorphisms in *miR‐100, miR‐146a, and miR‐210,* and susceptibility to childhood ALL. The result indicated that there was significant association between the polymorphisms in *miR‐100* (rs543412) and decreased susceptibility to childhood ALL. Besides, plasma miR‐100 was observed to be significantly elevated in ALL cases. Our results provided experimental evidence for the important role of miR‐100 in carcinogenesis of childhood ALL in both genetic and transcriptional levels. Further studies are needed to validate our findings and provide evidence to clarify the molecular mechanism.

## CONFLICT OF INTERESTS

The authors have no conflict of interest to report.

## Authors’ Contributions

Conceptualization and funding acquisition: Yao Xue and Yongjun Fang. Investigation and methodology: Xiaoyun Yang and Meiyun Kang. Data curation and formal analysis: Xiaoyun Yang. Sample collection: Shaoyan Hu and Jing Chen. Supervision and validation, and review and editing: Yongjun Fang. Writing—original draft: Yao Xue.
