# Supplementary material for: A genetic variant in miR‐100 is a protective factor of childhood acute lymphoblastic leukemia
Source: Cancer Med. 2019 Mar 7;8(5):2553–60. doi: 10.1002/cam4.2082 (PMC6536980; doi:10.1002/cam4.2082)
Supplement: Supplementary file 1 [file CAM4-8-2553-s001.docx]

Supplementary Table 1 Amplification primers used in the present study

|  | Upstream Primer | Downstream Primer |
| --- | --- | --- |
| rs543412 | GAACCAGGGAGCTCCCCGAAGGCG | GAACCAGGGTTGAATTGCACTCCGC |
| rs2910164 | TCAGGACAAAAGGTCTCCTTC | CCGAACTTGTGGTATTAGTCC |
| rs7395206 | GAAAAGCCGATGTGTATCCTC | TATACCTTCAGAGCCTGAGAC |
| miR-100 |  |  |
